# Recent Advances in the Treatment of Patients with Multiple Myeloma

**DOI:** 10.3390/cancers12123576

**Published:** 2020-11-30

**Authors:** Mario A. Legarda, María J. Cejalvo, Javier de la Rubia

**Affiliations:** 1 Hematology Department, University Hospital Doctor Peset, 46017 Valencia, Spain; legarda_mar@gva.es (M.A.L.); cejalvo_mar@gva.es (M.J.C.); 2 Hematology Department, Internal Medicine, School of Medicine and Dentistry, Catholic University of Valencia, 46017 Valencia, Spain

**Keywords:** multiple myeloma, autologous stem cell transplantation, consolidation, maintenance, relapsed refractory multiple myeloma, early relapse, late relapse, novel drugs, immunotherapy, car-t cells

## Abstract

**Simple Summary:**

The evolving data from trials assessing novel combinations as a part of the frontline and relapse treatment in transplant and non-transplant candidates have markedly improved the anti-myeloma efficacy of the different therapeutic regimens and improved patients’ prognosis. Current treatment objectives are focused to further improve the rate of complete remission, time to progression, progression-free survival and overall survival without increasing toxicity. Besides, different strategies are being developed in the elderly population as this group of patients requires a closer monitoring with individualized, dose-modified regimens to improve tolerability while maintaining their quality of life. This article presents a general description of the novelties of the whole treatment of multiple myeloma; from induction in the newly diagnosed patient through the role of hematopoietic stem cell transplantation and maintenance treatment until early and late relapses; including a section on recently approved drugs as well as novel drugs and immunotherapy in advanced stages of research.

**Abstract:**

In the past 20 years, few diseases have seen as great progress in their treatment as multiple myeloma. With the approval of many new drugs and the limited availability of clinical trials comparing head-to-head the different possible combinations, the choice of the best treatments at each stage of the disease becomes complex as well as crucial since multiple myeloma remains incurable. This article presents a general description of the novelties of the whole treatment of multiple myeloma, from induction in the newly diagnosed patient through the role of hematopoietic stem cell transplantation and maintenance treatment until early and late relapses, including a section on recently approved drugs as well as novel drugs and immunotherapy in advanced stages of research, and that will surely play a relevant role in the treatment of this devastating disease in the coming years.

## 1. Introduction

Multiple myeloma (MM) is a malignant B-cell disorder characterized by the clonal proliferation of plasma cells in bone marrow typically associated with overproduction of monoclonal proteins that accumulate in serum and urine. These alterations lead to extensive disease manifestations including anemia, hypercalcemia, immunosuppression, and end-organ damage such as renal impairment and bone lesions [1,2]. MM accounts for 1.8% of all cancers with an estimated 32,270 new cases and 12,830 deaths in the USA for 2020 [3], while in Europe there are more than 48,000 new cases and around 31,000 deaths each year [4]. 

Despite the enormous advances in the treatment of MM in the last decades and the availability of new drugs and their combinations, MM remains incurable, therefore using the best treatments available at each stage of the disease is of great importance, while epidemiological data encourage the continuous search for new treatments and therapeutic strategies to achieve prolonged survival with a good quality of life and perhaps achieve the so far elusive cure of the disease.

## 2. Treatment of Newly Diagnosed Multiple Myeloma

### 2.1. Front-Line Transplant Setting

**Figure 1:**
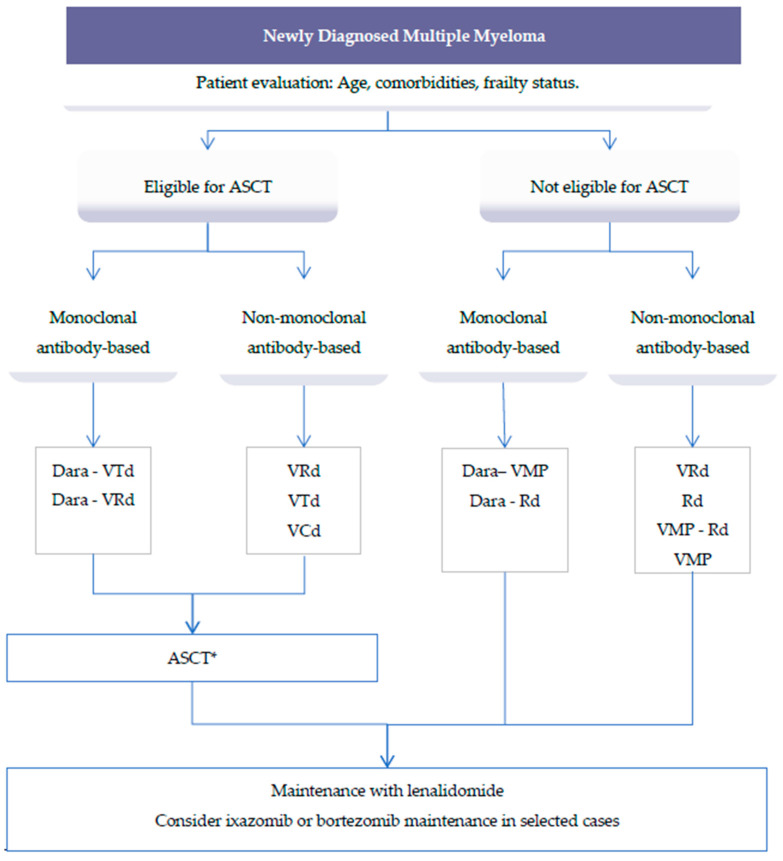
Suggested treatment options for newly diagnosed multiple myeloma (MM) patients. * Consider tandem ASCT in eligible high-risk patients. Abbreviations: ASCT: Autologous stem cell transplant; Dara: Daratumumab; VTd: Bortezomib, thalidomide, dexamethasone; VRd: Bortezomib, lenalidomide, dexamethasone; VCd: Bortezomib, cyclophosphamide, dexamethasone; VMP: Bortezomib, melphalan, prednisone; Rd: Lenalidomide, dexamethasone.

The treatment of multiple myeloma (MM) is rapidly evolving with the approval of multiple new drugs. In younger MM patients, the extended use of high-dose chemotherapy followed by autologous hematopoietic stem-cell transplantation (ASCT) is associated with high response rates and prolonged progression free survival (PFS) and overall survival (OS), and is still considered the standard of care in MM patients up to 70 years with newly diagnosed MM [5]. The suggested treatment options for newly diagnosed MM patients are presented in Figure 1.

The objective of the induction is to achieve high response and rates of minimal residual disease (MRD) with minimal toxicity to preserve stem cell harvesting. In fact, some authors have shown that MRD negative (<10^−6^) results measured by next-generation sequencing (NGS) either in the autograft product or in the bone marrow post-ASCT are associated to a significantly better PFS (96% at four years; *p*< 0.001) and OS (100% at four years; *p* = 0.04) than in MRD-positive patient [6].

Common regimens that have been tested in randomized trials in these groups of patients include bortezomib, thalidomide, dexamethasone (VTd), bortezomib, cyclophosphamide, dexamethasone (VCd), bortezomib, lenalidomide, dexamethasone (VRd), and, more recently, carfilzomib, lenalidomide, and dexamethasone (KRd) [7,8,9,10]. VTd showed a superior response rate compared with bortezomib and dexamethasone (Vd) alone in a phase III trial and was established as one of the standard of care regimens before ASCT in spite of its neurotoxicity [11]. VCd has less neurological toxicity compared to VTd, but a prospective comparison of four cycles of VTd vs. four cycles of VCd showed significant improvement in the thalidomide combination [12]. Aimed to reduce severe thalidomide-related side effects, VRd was tested as induction therapy for newly diagnosed, transplant-eligible patients with MM. This regimen is associated with a response rate of 89–97%, with 33–48% of patients achieving complete response (CR) and a progression-free survival (PFS) longer than four years and an overall survival (OS) at four years higher than 80% [13,14]. Furthermore, data from an integrated analysis of two studies from the Spanish myeloma group (GEM) including more than 500 patients with newly diagnosed MM (NDMM), and receiving pre-transplant induction with VTd or VRd showed that the rate of very good partial response (VGPR) or better and minimal residual disease (MRD)-negativity was significantly higher with VRd than with VTd (42% vs. 26%) [15]. Therefore, despite the absence of head-to-head comparisons, these results support the use of VRd as standard induction therapy for transplant-eligible patients with newly diagnosed MM.

With these data, the question that arises is if there is still room for improvement of induction therapy in young patients. Two alternatives have been recently tested in this field. One is the development of quadruplet therapies by adding the anti-CD38 monoclonal antibody daratumumab to VTd and VRd and the second is the combination of lenalidomide and dexamethasone including the second-in-line proteasome inhibitor (PI) carfilzomib (KRd).

The phase III clinical trial CASSIOPEA compared four cycles of pre-transplant induction with daratumumab plus VTd (D-VTd) or VTd followed by two cycles of consolidation after transplant in a series of 1085 transplant-eligible patients [16]. At day 100 after transplantation, results showed a higher stringent CR (sCR) among D-VTd recipients (29% vs. 20%, odds ratio [OR]: 1.60; *p* = 0.0010). CR or better was also higher with the quadruplet therapy (39% vs. 26%). Finally, 64% and 44% of patients achieved MRD-negativity with D-VTd and VTd, respectively. Median PFS was not reached in either group (hazard ratio [HR] 0.47; *p* < 0.0001). Therefore, this was first study showing the clinical benefit of D-VTd in this group of patients. VRd has also been combined with daratumumab (D-VRd) and compared with VRd in the GRIFFIN, phase II trial [17]. In this study, 207 patients were randomized to receive four cycles of induction with VRd ± D followed by ASCT, VRd ± D consolidation (two cycles), and lenalidomide ± D maintenance (26 cycles). With a median follow-up of 22.1 months, rates of sCR improved for D-VRd versus VRd (62.6% vs. 45.4%; *p* = 0.0177), as did rates of MRD-negativity in the intent-to-treat population (51.0% vs. 20.4%; *p* < 0.0001) with no new safety concerns. These data support the use of daratumumab-based quadruplet therapies as induction regimens in NDMM patients who are transplant-candidates. However, questions remain open, as it was observed than D-VTd and D-VRd did not improve the CR rates in the subgroup of patients with international staging system *for* myeloma (ISS) stage III disease or high-risk cytogenetics. 

Table 1 shows a summary of the most relevant findings coming from new induction trials in ASCT-eligible patients.

A second approach aimed to improve the results of current induction regimens has been the substitution of bortezomib by carfilzomib. In the phase II FORTE study, 281 patients were randomized to receive four cycles of induction with KRd followed by ASCT and four cycles of KRd consolidation (KRd-ASCT-KRd), versus the same approach with carfilzomib, cyclophosphamide, and dexamethasone (KCd) or 12 cycles of KRd without transplant (KR12). Results showed higher rates of VGPR or better of 89% and 87% in patients receiving KRd with or without transplant, respectively, vs. 76% in the KCd arm [19]. KRd was associated with more hematological G3–4 adverse events (13% vs. 9%), but no significantly increased cardiovascular toxicity (1% vs. 2%) compared to KCd [20]. In addition, data from the phase III, ENDURANCE trial comparing induction with KRd vs. VRd in more than 1000 patients who were not being considered for immediate ASCT and without high-risk MM have been recently available. At a median follow-up of nine months, the PFS was 34.6 months in the KRd group and 34.4 months in the VRd group (HR 1.04; *p* = 0.74). Median OS had not been reached in either group [21]

### 2.2. Role of Autologous Stem Cell Transplant (ASCT)

High dose melphalan followed by ASCT remains the standard of care for transplant-eligible patients with myeloma. Although, OS benefit was reported in some of the original trials, there was a consistent improvement in PFS [22,23,24,25,26]. As newer and more efficacious anti-MM regimens have been developed, some authors have reconsidered the role of front-line ASCT in younger patients with NDMM. In the previously mentioned FORTE trial [19], high and comparable rates of MRD-negativity were seen in the group of patients receiving KRd with or without transplant. However, in the multivariate regression analysis, patients in the KRd-ASCT-KRd group had a reduced risk of early progression vs. KRd12 (*p* = 0.021), particularly in patients with R-ISS stage 2 (*p* = 0.001) and 3 (*p* = 0.003) [18]. Overall, these results suggest that ASCT still plays a not yet replaceable anti-MM role. 

Finally, recent data support the role of tandem ASCT over single transplant to improve the poor prognosis of patients with R-ISS stage III (69% vs. 47% at three years; *p* = 0.009) and high-risk cytogenetics (74 % vs. 61%; *p* = 0.027), including those with del(17p) positivity (75% vs. 51%; *p* = 0.028) [27].

### 2.3. Post ASCT Treatment: Consolidation and Maintenance 

Despite the effectiveness of the therapeutic advances reached in transplant-candidates patients with NDMM, these patients continue to experience disease progression over time. Thus, additional strategies such as consolidation and maintenance therapy have been developed to prolong response and try to improve survival. Consolidation, defined as a short distinct course of treatment, is being used increasingly in routine practice. In addition, maintenance therapy, i.e., the administration of prolonged treatment for 12 or 24 months or until progression, is a standard approach after ASCT. 

Data from the previously mentioned GRIFFIN and CASSIOPEA trials showed an increase in CR or better of 24% and 25%, respectively after consolidation, supporting the use of this step in the global management of transplant-candidates [16,17]. These data, however, contrast with the mild increase in CR (<8%) and MRD-negativity rate (<3%) observed in the GEM2012 trial [13]. This finding could be partly explained by the difference in the duration of the induction regimen in those trials (six, 28-days cycle in the Spanish trial vs. 4, 21-days in the GRIFFIN trial and 28-days, pre-transplant induction cycles in CASSIOPEA). Finally, Stadmauer et al. compared in a three-arm phase III clinical trial, tandem ASCT followed by lenalidomide maintenance, ASCT plus four cycles of consolidation with RVd followed by lenalidomide, and ASCT followed lenalidomide only [28]. MM response assessments at one year did not show any difference between three arms (79.7%, 82.3%, and 76% in the tandem transplant, ASCT + consolidation, and ASCT + lenalidomide arms, respectively). Therefore, from our point of view, there are still insufficient data to determine if consolidation therapy improves long-term outcome and if it is routinely warranted for all patients or reserved for patients with failed VGPR or better after ASCT or for specific high-risk categories.

In terms of post-transplant maintenance, single-agent lenalidomide administered continuously until disease progression is considered the standard of care [29]. A meta-analysis including 1208 patients (605 patients in the lenalidomide maintenance group and 603 in the placebo or observation group) showed a significant benefit in terms of PFS (52.8 months vs. 23.5, respectively; HR, 0.48) in favor of lenalidomide-recipients. At a median follow-up time of 79.5 months, the median OS had not been reached vs. 86.0 months, respectively (HR 0.75; *p* = 0.001). In addition, the cumulative incidence rates of progression, death, or death because of myeloma were all higher with placebo or observation versus lenalidomide maintenance [30]. Recently, data of maintenance with the oral PI ixazomib from the TOURMALINE-MM3 study have been published. In this trial, 656 patients with NDMM and in partial response after single ASCT receive ixazomib or placebo on days 1, 8, and 15 in 28-day cycles for two years after transplantation. With a median follow-up of 31 months, there was a reduction of 28% in the risk progression or death with ixazomib versus placebo (median PFS 26.5 months vs. 21.3 months; HR 0.72; *p* = 0.0023) without new safety concerns [31]. Although results are less impressive than those achieved with lenalidomide monotherapy, ixazomib could represent a new alternative for some patients, particularly those experiencing intolerance or severe toxicity with lenalidomide. 

### 2.4. Front-Line Non-Transplant Setting 

Elderly patients are a heterogeneous and highly vulnerable population of patients that need an appropriate therapeutic approach ensuring keeping the best quality of life and apply supportive care measures from initial diagnosis to end of life. The arbitrary cut-off used to distinguish transplant-candidates vs. non-transplant candidates, based mostly on chronologic age (≥65−70 years), and the absence of a routine use of objective tools to clearly identify the frailty status of the patients (including, among others, comorbidities, disability, and dependence on help) are commonly pitfalls performed when deciding the best treatment for these group of patients. Therefore, we recommend the systematic application of a geriatric evaluation to improve clinical decision-making in older patients with hematological malignancies [32,33,34,35,36]. 

The GEM2010 trial phase 2 trial evaluated a sequential vs. alternating administration of nine cycles of VMP and nine cycles of Rd as front-line treatment in elderly patients (*n* = 233). Both arms yielded similar anti myeloma efficacy, both in terms of CR (42% vs. 40%), median PFS (32 months vs. 34 months; *p* = 0.65), and three-year OS (72% vs. 74%; *p* = 0.63) [37]. Notably, MRD-negative status surpasses the prognostic value of CR achievement for PFS and OS across the disease spectrum, regardless of the type of treatment or patient risk group [38]. 

VRd has also shown significant efficacy in the setting of NDMM in these patients. In the randomized, open-label, phase 3 SWOG S0777 trial, 525 patients not planned to undergo front-line transplant, were randomly assigned (1:1) to receive initial treatment of bortezomib with lenalidomide and dexamethasone (VRd, 264 patients) or lenalidomide and dexamethasone (Rd, 261 patients). At a median follow up of 84 months, median PFS was 41 months for VRd and 29 months for Rd (*p* = 0.003). Median OS for VRd had not been reached and was 69 months for Rd (*p* = 0.0114) [39]. 

Results of the multicenter, open-label, phase 3, randomized controlled ENDURANCE trial (E1A11) have been recently published. This study compared front-line induction with Krd vs. VRd in 1087 patients with NDMM who were ineligible for, or did not intend to have, immediate ASCT. At a median follow-up of nine months, the median PFS was 34.6 months in the KRd group and 34.4 months in the VRd group (HR: 1.04; *p* = 0.74). Median overall survival was not reached in either group. According to authors’ conclusions, KRd regimen did not show significant advantage and was associated with more toxicity. Therefore, VRd regimen remains the standard of care for induction therapy for patients with standard-risk and intermediate-risk NDMM, and is a suitable treatment backbone for the development of combinations of four drugs [21].

Precisely, several ongoing trials are investigating daratumumab-based combinations in elderly patients. Recently, the interim analysis of a phase 3 study (*n* = 737) comparing Rd±D were published [40]. With a median of follow-up of 28 months, disease progression or death was 26.4% in the daratumumab group and 38.8% in the control group. The percentage of patients with a CR or better was 47.6% in the daratumumab group and 24.9% in the control group (*p* < 0.001). A total of 24.2% of the patients in the daratumumab group, as compared with 7.3% of the patients in the control group, had results below the threshold for MRD with a sensitivity of 1 × 10^−5^ (*p* < 0.001) [40] (Table 2). The phase 3 study (*n* = 706) comparing daratumumab plus VMP (Dara-VMP) vs. VMP showed a significant longer PFS with D-VMP (HR 0.42; *p* = 0.0001) [41,42]. In addition, with a median of follow-up of 40.1 months, there was significant benefit in OS in favor of the quadruplet regimen (36-month rate of OS, 78.0%) vs. VMP (67.9%) HR: 0.60; *p* = 0.0003).

The selection of treatment in elderly patients should also consider the risk of toxicity and the capability to tolerate treatment, since advanced age and the occurrence of severe adverse events may negatively affect survival. In this regard, a recent meta-analysis showed favorable efficacy outcomes for daratumumab-based regimens vs. other relevant frontline options in most of the subgroups analyzed [43]. However, the addition of daratumumab did not seem to overcome the poor prognosis of high-risk patients. Therefore, additional trials testing multi-drug combinations including IMiD, second-generation PI are needed to evaluate and, potentially, improve the outcome of elderly patients with high-risk disease.

## 3. Treatment of Relapsed Multiple Myeloma

In MM it is well know that the PFS duration decreases after each relapse, making of paramount importance the election of treatment for relapsed refractory multiple myeloma (RRMM) [44]. The choice of the most appropriate treatment for these patients should be based on multiple factors including duration and type of response to prior treatments, prior toxicities, performance status, comorbidities, and cytogenetic risk. As a rule, in the management of RRMM it is generally accepted that rescue regimens should include drugs with different mechanisms of action to those previously administered, particularly in patients with short PFS duration or suboptimal response [45]. In this field, several clinical trials have confirmed that combination of three drugs are associated to better results in terms of quality and duration of response (DoR) when compared with a two-drug regimen [46]. Therefore, the recommendation is to treat with a triplet chosen according to refractoriness to previous treatments so that the new regimen includes two drugs to which the patient is not refractory.

### 3.1. Treatment of Early Relapse

We refer to early relapse understood as a relapse after one or two lines of treatment and even a third line when optimal therapy was not received in the first lines. Over the last years, several trials with three drug combinations, including lenalidomide have been tested as rescue therapies in patients with RRMM [47,48,49,50,51,52,53]. All these trials consistently showed superior clinical activity of the triplet therapy when compared against lenalidomide-dexamethasone alone and, therefore, they became standard regimens for this group of patients. In addition, PI-based combinations have been also evaluated in this setting, including patients who were previously exposed to lenalidomide or were lenalidomide-refractory [54,55,56,57]. Overall, these regimens showed also impressive anti myeloma activity and improved results over standard strategies. All these rescue combinations are widely used in clinical practice and their results have been extensively reviewed elsewhere [58,59,60,61], thus they will not be discussed here (Table 3).

**Figure 2:**
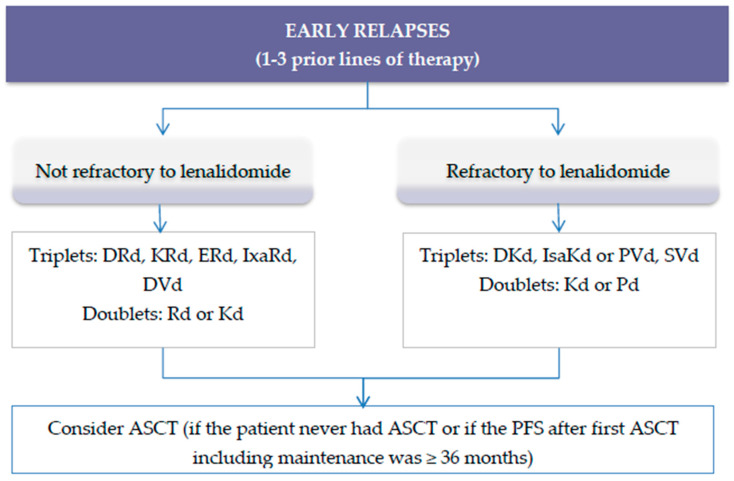
Suggested algorithm to treat patients with RRMM and 1−3 prior lines of therapy. Abbreviations: DRd: Daratumumab, lenalidomide, dexamethasone; KRd: Carfilzomib, lenalidomide, dexamethasone; ERd: Elotuzumab, lenalidomide, dexamethasone; IxaRd: Ixazomib, lenalidomide, dexamethasone; DVd: daratumumab, bortezomib, dexamethasone; Rd: Lenalidomide, dexamethasone; Kd: Carfilzomib, dexamethasone; IsaKd: Isatuximab, carfilzomib, dexamethasone; DKd: Daratumumab, carfilzomib, dexamethasone; PVd: Pomalidomide, bortezomib, dexamethasone; Pd: Pomalidomide, dexamethasone; SelVd: Selinexor, bortezomib, dexamethasone; ASCT: Autologous stem cell transplant.

However, as lenalidomide has been progressively incorporated into the front-line regimens used for the treatment of patients with NDMM, these “first generation” combinations become no longer useful in an important number of patients. Because most of the patients receive lenalidomide front-line until disease progression, per definition, the majority of them become lenalidomide-refractory at the time of relapse and render lenalidomide-based therapies useless. Figure 2 shows the suggested algorithm to treat patients with early RRMM.

Finally, as it has been previously mentioned, the approval of daratumumab as combination therapy in patients with newly diagnosed transplant-ineligible (Dara-VMP, DRd) and transplant eligible (D-VTd, D-VRd) patients will expand the range of MM treatment settings in which daratumumab is an option. This highlights a new and important challenge in MM therapy in the future for finding strategies that improve the survival of CD38 monoclonal antibody-refractory patients.

#### Second Generation Regimens for Relapsed Refractory Multiple Myeloma (RRMM)

Therefore, many newer lenalidomide-sparing regimens aimed to skip the len-refractoriness status have been recently tested in numerous clinical trials (Table 4) [62,63,64,65,66,67].

One important limitation of all of them, however, is that the comparator has been a two-drug regimen, despite it is generally accepted that doublets are suboptimal approaches to rescue patients with RRMM. Nevertheless, and although in many of these new therapies results are still preliminary, these second-generation triplets have consistently showed a high clinical activity in terms of response and PFS duration.

Among them, the association of monoclonal antibodies (MoAbs) targeting CD38 (daratumumab and isatuximab) with carfilzomib has been extensively studied. There are two phase 3 trials comparing carfilzomib and dexamethasone (Kd) vs. Kd with daratumumab (CANDOR) [62] or isatuximab (IKEMA) [63] in patients with RRMM and a median of two prior lines of therapy. Both trials have a similar design, and the proportion of lenalidomide-refractory patients (32−33%) was also similar. The main differences between both trials are a greater proportion of patients with high risk cytogenetic in the Isa-Kd study (33% vs. 15%) and a slightly more heavily pretreated population in the IKEMA study (18% of patients received more than three prior lines, versus 0% in the CANDOR trial). From the efficacy point of view, both triplets showed a superior clinical activity over Kd, with a median PFS not reached after a median follow-up of 17 and 20.7 months in the CANDOR and IKEMA trial, respectively. Similarly, the overall response rate (ORR) was superimposable in both trials (Table 4) with a slightly higher proportion of CR with the Isa-Kd combination (40% vs. 29%). From the safety point of view, the most frequent hematological ≥G3 side effects were neutropenia (19% with Isa-Kd and 8% with Dara-Kd) and thrombocytopenia (30% and 24%, respectively). Respiratory infections were also common with the anti-CD38 based therapies, with 24% of the patients in the IKEMA and 13% in the CANDOR trials developing ≥G3 pneumonia. Finally, the incidence of severe cardiac failure was low (≈4%) and similar in both studies [62,63].

Another regimen tested in lenalidomide treated patients is the combination of pomalidomide, bortezomib, and dexamethasone (PVd). In the phase 3 OPTIMISMM trial, 559 RRMM patients with a median of 2 (range, 1−3) prior treatment regimens, were randomly assigned to receive PVd vs. Vd [67]. Most of the patients (≥69%) were refractory to lenalidomide with all of them previously exposed and (≥75%) also exposed to bortezomib. At a median follow-up of 15.9 months, the median PFS was better for the PVd group (11.2 months vs. 7.1 months with Vd, HR 0.61; *p* < 0.0001). ORR and VGPR or better were also higher with PVd than with Vd, 82.2% vs. 50% (odds ratio [OR] 5.02; *p* < 0.0001) and 52.7% vs. 18.3% (OR 5.0; *p* < 0.0001). The most commonly observed G≥3 adverse events (AE) were neutropenia (42% vs. 9%), thrombocytopenia (27% vs. 29%), and infection (31% vs. 18%) in the PVd vs. Vd groups, respectively. 

Alternatively, if the patient had not received an ASCT in previous lines, or if had a good response first ASCT, high-dose chemotherapy followed by ASCT can be also considered an option [68,69,70]. Unfortunately, myeloma cells still retain the ability of developing mechanisms of resistance that renders most of these new combinations inefficacious over time. Therefore, a “third-generation” of novel immunotherapies have entered into scene in the rapidly moving field of myeloma therapy, and for sure will play a relevant role in the forthcoming years.

### 3.2. Late Relapses

Newer regimens have been designed to improve clinical results in patients suffering several relapses (Table 5) [71,72,73]. Among them, the combination of isatuximab, pomalidomide, and dexamethasone (Isa-PD) has been the only combination tested in a phase 3 trial (ICARIA). In this study, Attal et al. compared pomalidomide and dexamethasone (Pd) versus Isa-Pd in 307 patients. The median (range) of prior regimens was 3 (2−4), including lenalidomide and PI in all cases. Also, a significant number of patients showed refractoriness to IMiDs (94%) and PI (77%) [71]. More patients responded in the Isa-Pd group vs. Pd group (60% vs. 35%; *p* < 0.0001) and after a median follow-up of 11.6 the median PFS was 11.5 months vs. 6.5 months (HR, 0.59; *p* = 0.001) in the Isa-Pd and in the Pd group, respectively with median OS not reached in either group. Dimopoulos et al. reported the results of the randomized phase 2, ELOQUENT 3 trial comparing elotuzumab (anti SLAMF7 monoclonal antibody) with Pd (Elo-Pd) versus Pd in 117 patients with a median (range) of 3 (2−8) prior treatment regimens [72]. Most of the patients were refractory to lenalidomide (90%) and bortezomib (78%). In this study ORR was better with Elo-Pd (53% vs. 26%, respectively; OR, 3.25). At a minimum follow-up of 9.1 months, the median PFS was 10.3 months in the Elo-Pd vs. 4.7 months in the Pd arm, with a HR of 0.54 (*p* = 0.008). Median OS was not reached in any group.

The combination of daratumumab and Pd (Dara-Pd) has also been tested in the multi-arm, open-label, nonrandomized, phase 1b EQUULES trial [73,74]. This study included heavily pretreated patients with a median (range) of 4 (1−13) prior treatment regimens. Besides, there was a high proportion of patients refractory to lenalidomide (89%), bortezomib (71%), and carfilzomib (30%). At a median follow-up of 24.7 months, the overall response rate (ORR) was 66% and the VGPR or better was 48%. Median PFS was 9.9 months and the median OS was 25.1 months. Dara-Pd has also shown promising results in the phase II MM-014 trial including 112 less heavily pretreated patients, 75% of whom were refractory to lenalidomide [75]. With a median of follow-up of 8.2 months, the ORR was 75% in lenalidomide-refractory patients, and the 9-month PFS rate in these patients was 86.3%. 

**Figure 3:**
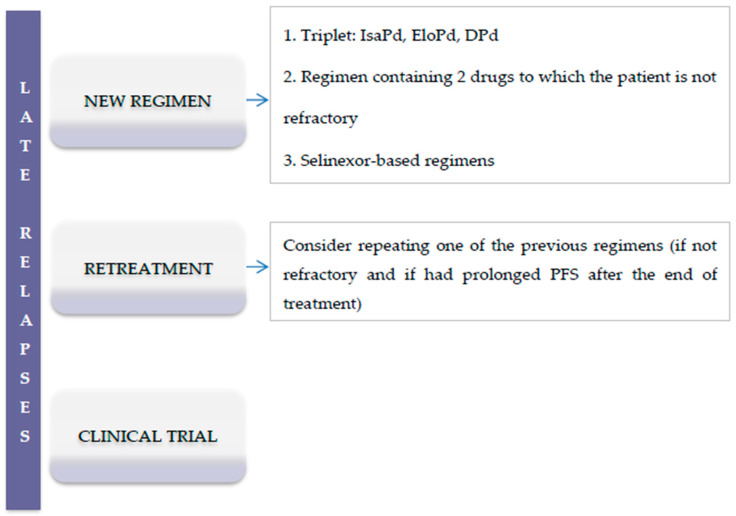
Suggested treatment options for RRMM patients in advanced phases of the disease (>3 lines of therapy). Abbreviations: IsaPd: Isatuximab, pomalidomide, dexamethasone; EloPd: Elotuzumab, pomalidomide, dexamethasone; DPd: Daratumumab, pomalidomide, dexamethasone; PFS, progression-free survival.

Other regimens that have been tested in patients with advanced phases of the disease include carfilzomib-Pd [76], ixazomib-Pd [77], and the combination of the oral histone deacetylase inhibitor panobinostat with carfilzomib [78]. Results of these trials are shown in Table 6 and in the Figure 3 are shown the suggested treatment options for RRMM patients in advanced phases of the disease.

## 4. New Generation Therapies and Future Insights

As natural history of RRMM continue treatments are less effective and for patients whose disease has become refractory to PIs, IMiDs, and CD38 MoAbs, the outcome is very poor with a recent study indicating a median OS of 5.6 months only [79]. This fact strongly supports the necessity of the incorporation of novel drugs for the treatment of RRMM. In this field, new treatments such as selinexor has been recently approved for use in RRMM. There are also new promising molecules in advanced stages of research such as melflufen, iberdomide, and venetoclax. The final role of these drugs in the treatment of RRMM still needs to be defined. However, the greatest hopes are placed on a third generation of rescue therapies that includes conjugated antibodies, bispecific antibodies, and chimeric antigen receptor T-cell therapy (CAR-T).

### 4.1. Novel Drugs

#### 4.1.1. Selinexor

Selinexor is an orally bioavailable, potent and selective inhibitor of nuclear export, slowly reversible that blocks specifically exportin 1. This protein participates in the nucleus–cytoplasm protein traffic and its blockade induce apoptosis by means of high nuclear concentrations of apoptotic proteins [80]. The combination of selinexor and dexamethasone was tested in the phase 2b STORM trial that included 122 heavily pretreated patients with a median (range) of 7 (3−18) prior lines of treatment [81]. Every patient was refractory to PIs, IMiDs, and CD38 MoAbs and high-risk cytogenetic abnormalities were present in 53% of patients. The ORR was 26% and minimal response or better was achieved in 39% of patients. Median PFS was 3.7 months and median OS was 8.6 months. In patients who reached at least minimal response the OS was 15.6 months. The results of this trial granted accelerated FDA approval to selinexor in combination with dexamethasone for adult patients with RRMM who have received at least four prior therapies and whose disease is refractory to at least two PIs, at least two IMiDs, and a CD38 MoAb [82]. There are several phase 3 trials testing new selinexor-based triplet regimens that may broad the use of this new drug in less heavily pretreated patients [64,83].

#### 4.1.2. Melflufen

Melphalan flufenamide (melflufen) is a novel lipophilic peptide-conjugated alkylator that is rapidly taken up by myeloma cells due to its high lipophilicity and through aminopeptidases action is cleaved into melphalan and p-Fluorophenylalanine, that leads to melphalan accumulation and consequently DNA damage and apoptosis [84]. In the phase 2 HORIZON trial, Mateos et al. studied 154 heavily pretreated patients with a median (range) of 5 (2−12) prior lines of treatment, most of them (71%) had triple-class RRMM [85,86]. Melflufen was administered at a dose of 40 mg (IV on day 1 of each 28-days cycle) with weekly dexamethasone until disease progression or unacceptable toxicity. Among the evaluable patients the ORR was 28% and with a median follow up of 15.3 months, the median PFS was 4.2 months. Several other clinical trials including melflufen are underway that will help to definitively define the role of this drug in RRMM patients.

#### 4.1.3. Iberdomide

Iberdomide (CC220) is a new development as a cereblon-targeted drug that has greater antiproliferative effect in vitro than pomalidomide and lenalidomide. In addition, a synergistic effect is observed in the combination of this drug daratumumab, bortezomib, and dexamethasone [87]. The first clinical results evaluating iberdomide plus dexamethasone were presented by Lonial et al. [88] in a phase 1b/2a trial that included 58 heavily pretreated patients with a median of 5 (range, 2−12) prior lines of therapy. Results showed a 31% ORR and the most common observed ≥ G3 AE were neutropenia (26%) and thrombocytopenia (11%) with a discontinuation rate due to AEs of 5%. The study is ongoing including patients receiving iberdomide in combination with bortezomib or daratumumab.

#### 4.1.4. Venetoclax

It is an orally bioavailable selective inhibitor of BCL-2, an antiapoptotic protein whose overexpression is related to tumor cell survival and resistance to chemotherapeutics. A total of 291 patients with 1−3 prior lines of therapy were 2:1 randomized to receive venetoclax with bortezomib and dexamethasone or Vd in the phase 3 randomized BELLINI trial. At a median follow-up of 28.6 months, there were 64 (33%) deaths in the venetoclax arm vs. 24 (25%) in the placebo arm, PFS was 23.2 months in the venetoclax arm vs. 11.4 in the placebo arm (HR 0.60), and OS of 33.5 months with venetoclax vs. not reached with placebo (HR 1.46). Interestingly, in prespecified analysis of 35 patients with t(11;14) the median PFS was not reached with venetoclax versus 9.5 months with placebo (HR 0.11; *p* = 0.0040). Response rates and MRD negativity rates were also higher with venetoclax in this subgroup [89]. Some studies suggest that part of the lack of effectiveness of venetoclax may be mediated by the compensatory increase in the anti-apoptotic protein MCL-1, so they have tried to combine venetoclax with a selective MCL-1 inhibitor with good preclinical results [90]. Additionally other studies combining venetoclax with carfilzomib and bortezomib are ongoing with encouraging preliminary results [91,92]. While we wait for the final data of this trial, venetoclax, bortezomib, dexamethasone should be considered as the most appropriate treatment for MM patients with t(11;14).

### 4.2. Immunotherapy

#### 4.2.1. Belantamab Mafodotin

This novel conjugate drug combines a humanized, afucosylated monoclonal antibody directed to the B-cell maturation antigen (BCMA) with the microtubule-disrupting agent, monomethyl auristatin F (MMAF) [93]. Lonial et al. carry out the phase 2, DREAMM-2 study including 196 RRMM patients that were randomly assigned (1:1) to receive 2.5 mg/kg (*n* = 97) or 3.4 mg/kg (*n* = 99) belantamab mafodotin intravenously every three weeks. The patients were heavily pretreated with a median (range) of 7 (3−21) prior lines of treatment, most of them refractory to PI, lenalidomide, pomalidomide, and daratumumab. ORR was 31% for the 2.5 mg/kg cohort and 35% in the 3.4 mg/kg cohort. At median follow-up period of 13 months the median PFS was 2.8 months (95% CI, 1.6–3.6) vs. 3.9 months (95% CI, 2.0–5.8), respectively and median OS was 13.7 months (95%CI, 9.9–NR) vs. 13.8 months (95% CI, 10–NR) respectively. The median DoR estimate was 11 months (95% CI, 4.2–NR) in the 2.5 mg/kg group and 6.2 months (95% CI, 4.8–NR) in the 3.4 mg/kg group. With these results, the recommended dose for belantamab was 2.5 mg/kg. The most common ≥G3 AE observed in the 2.5 mg/kg cohort of patients were keratopathy (27%), thrombocytopenia (20%), anemia (20%), and pneumonia (4%) [94,95]. Belantamab mafadotin has been recently approved in the US and EU for the treatment of RRMM [96].

#### 4.2.2. Bispecific Antibodies

Bispecific T-cell engagers (BiTEs) are artificial antibodies that produce an immune interaction through two specific domains directed to a T-cell and to a cancer cell, in the case of multiple myeloma the BiTEs are directed against BCMA and CD3 so far. Through this junction T cells are activated, and in proximity to MM cells are able to destroy them. CC-93269 is a BiTE with bivalent, specific BCMA binding and a flexible linker to CD3ε-binding Fab domains. The phase 1 trial for CC-93269 reported the inclusion of 30 RRMM patients with progressive disease after ≥3 previous lines of therapy, the safety analysis demonstrated cytokine release syndrome (CRS) in 77% of patients (3% ≥ G3) as the main AE. Results showed a high ORR of 88.9% in the higher dosing cohorts, suggesting that even in a heavily pretreated patient population, BiTE technology can effectively engage the immune system to kill MM cells, indicating that this could be an exciting treatment approach for patients with MM [97]. A larger phase II study is currently underway to further evaluate CC-9326 therapy. There are other anti-BCMA BiTEs also in development. The BiTE AMG-420 phase 1 trial has been published recently, including 42 patients with a median of 3.5 (range, 1−10) of prior regimens. Single-agent AMG-420 was administered at doses ranging from 0.2 to 800 µg/d mg/d. The maximum tolerated dose was 400 µg/d, the ORR was 31%, and at that dose the ORR response rate was 70%. However, this agent is not without adverse events, particularly infections and neuropathy [98]. Another anti-BCMA BiTE AMG-701 is under clinical trial with the same target but with significantly longer half-life for which it will replace AMG-402 in future developments.

#### 4.2.3. CAR-T Cells

CAR-T cell therapy is one of the most exciting new developing treatments for RRMM. It uses autologous T-cells modified in vitro to engage MM cell antigens. After encouraging results in the phase 1 trial [99] of Idecabtagene vicleucel (ide-cel), a BCMA-targeting CAR T-cell construct, the initial results of phase 2 KarMMa study have been published recently [100]. In this trial, 140 patients with RRMM, with more than three prior lines of treatment and previously exposed to PI, IMiD, and CD38 MoAb were enrolled and 128 were treated with a dose of 150−450 × 106 CAR-T cells. The reported median follow-up was 13.3 months observing an ORR of 73.4% with 33% CR rate, and a PFS of 8.8 months. The most common AE were cytopenias (97%) and CRS (84%), mostly ≤G2, with only 7% ≥G3. Another promising CAR-T cell is JNJ-4528/LCAR–B38M, a construct with a 4-1BB costimulatory domain and two BCMA targeting domains directed against two distinct BCMA epitopes, of which we have initial results from two trials [101]. The phase 1b/2 CARTITUDE trial assessed the safety and efficacy of the anti-BCMA CAR T-cell construct JNJ-4528 which is identical to LCAR–B38M [102]. In this trial, 29 RRMM heavily pretreated patients with a median number of 5 (range, 3−18) prior regimens, received JNJ-4528 infusion (0.5−1 × 106 cells). With a median follow-up of nine months, the ORR was 100% with 76% stringent CR, and 21% VGPR and PFS was 93%. The AE associated with JNJ-4528 were similar to those seen with other CAR T-cell constructs (CRS and neurotoxicity), although rates of G3/4 events were low [103]. 

Finally, the results of long-term follow-up of the phase 1 LEGEND-2 trial have also been reported. In this study, 57 RRMM less heavily pretreated patients with a median of 3 (range, 1−9) prior regimens, received infusion of LCAR- with dosing of 0.07−2.1 × 106 cells/kg. Overall, 78% of patients achieved a VGPR or better and the median DoR was 27 months, the median PFS was 19.9 months, and the median OS was 36.1 months [104]. 

There are other CAR-Ts in clinical and preclinical research targeting BCMA and many other antigens [105,106], so we may expect encouraging results in the near future, however there is still no plateau in survival in contrast to that seen with CAR-T cells treatment in other hematologic malignancies.

## 5. Conclusions

The results of this review reflect the rapid moving field in MM therapy and the exciting new treatments that will be available in the future. In the transplant-setting are particularly interesting the results achieved by the new quadruplet therapies including monoclonal antibodies, D-VTd and D-VRd. These regimens will probably be the corner stone of the newer treatment algorithms recommended by most of the international cooperative groups for younger patients with NDMM. The introduction of a monoclonal-based therapy has also become a standard front-line therapy in non-transplant eligible patients. D-VMP and particularly DRd regimens are associated with impressive results, rivalling in terms of duration and quality of responses with those obtained with the classic triplets in younger patients.

The development of lenalidomide-sparing regimens is one of the most burning points in the RRMM setting. As has been mentioned, the progressive growing number of lenalidomide-refractory patients at the time of relapse has boosted the development of several regimens focused on this patient population and, like with the front-line therapies, the use of monoclonal antibodies are part of most of these combinations (DKd, IsaKd, DPd, EloPd, IsaPd). However, the low number of patients in some of these studies, the short follow-up, and absence of head-to head comparisons make the decision-making process a difficult task for most physicians.

Likewise, there are still unmet medical needs in distinct patient subgroups. Renal impairment (RI) is one of the most common complications of symptomatic MM, affecting 20–30% of patients at the time of diagnosis and around 10% of them require haemodialysis, with a negative impact on prognosis [107,108]. Most trials performed in MM excluded patients with RI and though the use of novel anti myeloma agents have resulted in a substantial increase in the survival of these patients, RI remains one of the most challenging situations in clinical practice [109,110]. Besides, the management of MM patients presenting with high-risk cytogenetic negatively affect both PFS and OS both in patients with NDMM and RRMM. Recent treatment strategies combining PI with lenalidomide/pomalidomide and double autologous stem cell transplant have shown promise for HR cytogenetic diseases. Careful analysis of cytogenetic subgroups in trials comparing different treatments remains an important goal. Cross-trial comparisons may provide insight into the effect of new drugs in patients with cytogenetic abnormalities. However, to achieve this, consensus on definitions of analytical techniques, proportion of abnormal cells, and treatment regimens is needed.

Finally, the irruption of the new immunotherapy therapies, including BiTEs, CAR-T, and conjugated antibodies, has marked a turning point in the treatment of patients with MM. Though with low number of patients and still short follow-up, these approaches will play an essential role in the treatment of MM in the next years. In this setting, we believe that the design of trials including these therapies in earlier phases of the disease, taking advantage of a better-preserved immune system in the patient, will have far-reaching implications. 

With all these therapeutic armamentaria in the horizon, one of the most important efforts of the scientific community in the treatment of MM in the near future will be how to best combine all these agents early in the disease course to improve survival while reducing toxic effects. Together, these therapies should lead to higher response rates, more durable DoR, less toxicity, and prolonged survival for patients, making MM an increasingly treatable disease. Patients deserve no less. 

## Figures and Tables

**Table 1:** Results of induction/consolidation therapies in ASCT-eligible patient.

Treatment		No. of Cycles	Post-Induction Response (%)			Post-Consolidation Response (%)			
Regimen	No. of Patients	Induction/Consolidation	≥VGPR	≥CR	s-CR	≥VGPR	≥CR	s-CR	Trial Name
VTd 28 d	542	4/2	56	9	6.5	78	26	20	CASSIOPEA [16]
D-VTd 28 d	543	4/2	65	14	7	83	39	29	
VRd 21 d	102	4/2	57	13	7	73	42	32	GRIFFIN [17]
D-VRd 21 d	99	4/2	72	19	12	91	51.5	42	
VRd 21 d	350	3/2	47	-	-	78	-	-	IFM 2009 [14]
VRD 28 d	458	6/2	67	33	-	75.5	50	-	GEM 2012 [15]
KRd 28 d	158	4/4	67	34	8	87	62	41	FORTE [18]

Abbreviations: ASCT: Autologous stem cell transplant; VGPR: Very good partial response; CR: Complete response; PR: Partial response; SD: Stable disease.

**Table 2:** Randomized trials of induction therapies in ASCT-ineligible patients.

Treatment	No. of Patients	ORR (%)	≥CR	≥VGPR	PR	SD	Trial Name
Dara-Rd	368	92.9	47.6	79.3	13.6	3	MAIA [40]
Rd	369	81.3	24.9	53.1	28.2	15.2	
Dara-VMP	350	91	42.6	71.1	19.7	5.7	ALCYONE [41]
VMP	356	74	24.4	41.1	24.2	21.3	
VRd	264	90.2	24.2	74.9	15.3	7	SWOG S0777 [39]
Rd	261	78.8	12.1	53.2	25.6	16.4	

Abbreviations: ASCT: Autologous stem cell transplant; VGPR: Very good partial response; CR: Complete response; PR: Partial response; SD: Stable disease.

**Table 3:** Randomized clinical trials of first generation regimens in relapsed refractory multiple myeloma (RRMM).

Treatment	No. of Patients	Overall Response %	PFS—HR	PFS—Months	OS (Months)	Grade ≥ 3 AE	Median (Range) Prior Lines of Therapy	Trial Name
DRd vs. Rd	569	93 vs. 76	0.44	44.5 vs. 17.5	NR	90% vs. 81%	1 (1−11)	POLLUX [47]
KRd vs. Rd	792	87 vs. 67	0.69	26.3 vs. 17.6	48.3 vs. 40.4	87% vs. 83%	2 (1−4)	ASPIRE [49]
EloRd vs. Rd	646	79 vs. 66	0.71	19.4 vs. 14.9	48 vs. 40	77% vs. 68%	2 (1−4)	ELOQUENT 2 [50,51]
IxaRd vs. Rd	722	78 vs. 72	0.74	20.6 vs. 14.7	NR	74% vs. 69%	1 (1−3)	TOURMALINE MM1 [52]
DVd vs. Vd	498	85 vs. 63	0.31	16.7 vs. 7.1	NR	NA	2 (1−9)	CASTOR [56]
Kd vs. Vd	929	77 vs. 63	0.53	18.7 vs. 9.4	47.8 vs. 38.8	82% vs. 71%	2 (1−2)	ENDEAVOR [55]

Abbreviations: PFS: Progression free survival; HR: Hazard ratio; OS: Overall survival; AE: Adverse events; NR: Not reached; NA: Not available; DRd: Daratumumab, lenalidomide, dexamethasone; Rd: Lenalidomide, dexamethasone; KRd: Carfilzomib, lenalidomide, dexamethasone; EloRd: Elotuzumab, lenalidomide, dexamethasone; IxaRd: Ixazomib, lenalidomide, dexamethasone; DVd: Daratumumab, bortezomib, dexamethasone; Vd: Bortezomib, dexamethasone; Kd: Carfilzomib, dexamethasone.

**Table 4:** Recent lenalidomide-free randomized trials in RRMM.

Treatment	No. of Patients	Overall Response %	PFS—HR	PFS—Months	OS—Months	Grade ≥ 3 AE	Median (Range) of Prior Lines of Therapy	Trial Name
Isa-Kd vs. Kd	302	87 vs. 83	0.53	NR vs. 19.1	NR	77% vs. 67%	2 (1−4)	IKEMA [63]
DKd vs. Kd	466	93 vs. 86	0.63	NR vs. 15.8	NR	82% vs. 74%	2 (1−2)	CANDOR [62]
PVd vs. Vd	559	82 vs. 50	0.61	11.2 vs. 7.1	NR	NA	2 (1−3)	OPTIMISMM [67]
SelVd vs. Vd	402	76 vs. 62	0.7	13.9 vs. 9.5	NR vs. 25	NA	1 (1−3)	BOSTON [64]
PanVd vs. Vd	768	61 vs. 55	0.63	12 vs. 8	33.6 vs. 30.4	96% vs. 82%	1 (1−3)	PANORAMA [65]

Abbreviations: PFS: Progression free survival; HR: Hazard ratio; OS: Overall survival, AE: Adverse events; NR: Not reached; NA: Not available; Isa-KD: Isatuximab, carfilzomib, dexamethasone; Kd: Carfilzomib, dexamethasone; DKd: Daratumumab, carfilzomib, dexamethasone; PVd: Pomalidomide, bortezomib, dexamethasone; SelVd: Selinexor, bortezomib, dexamethasone; PanVd: Panobinostat, bortezomib, dexamethasone.

**Table 5:** Clinical trials in patients with ≥ 3 lines of treatment.

Treatment	No. of Patients	Overall Response %	PFS—HR	PFS—Months	OS—Months	Grade ≥ 3 AE %	Median (Range) of Prior Lines of Therapy	Trial Name
IsaPd vs. Pd	307	60 vs. 35	0.59	11.5 vs. 6.5	NR	NA	3 (2−4)	ICARIA [69]
EloPd vs. Pd	117	53 vs. 26	0.54	10.3 vs. 4.7	NR	57 vs. 60	3 (2−8)	ELOQUENT 3 [70]
DPd	103	66	NA	9.9	25.1	99	4 (1−13)	EQUULEUS [71]

Abbreviations: PFS, Progression free survival; HR, Hazard ratio; OS, Overall survival, AE, Adverse events; NR, Not reached; NA, Not available; IsaPd: Isatuximab, pomalidomide, dexamethasone; Pd: Pomalidomide, dexamethasone; EloPd: Elotuzumab, pomalidomide, dexamethasone; DPd: Daratumumab, pomalidomide, dexamethasone.

**Table 6:** Phase I studies of alternative regimens for late relapses in MM.

Treatment	No. of Patients	Overall Response %	PFS—Months	OS—Months	Grade ≥ 3 AE	Median (Range) of Prior Lines of Therapy
KPd [76]	32	50	7.2	20.6	63%	6 (2−12)
IxaPd [77]	31	48	8.6	NR	74%	2 (1−5)
PanKd [78]	30	57	8	23	63%	4 (1−8)

Abbreviations: PFS: Progression free survival; OS: Overall survival, AE: Adverse events; NR: Not reached; KPd: Carfilzomib, pomalidomide, dexamethasone; IxaPd: Ixazomib, pomalidomide, dexamethasone; PanKd: Panobinostat, carfilzomib, dexamethasone.
